# Co‐Design of an Ecosystem of Services to Support Veteran Well‐Being and Reduce Excessive Alcohol Consumption

**DOI:** 10.1111/hex.70262

**Published:** 2025-04-17

**Authors:** Julia Carins, Ann‐Marie Kennedy, Ekant Veer

**Affiliations:** ^1^ Department of Tourism and Marketing Griffith University Brisbane Australia; ^2^ Department of Management, Marketing and Tourism University of Canterbury Christchurch New Zealand

**Keywords:** alcohol, co‐design, qualitative, social marketing, veterans, well‐being

## Abstract

**Background:**

Alcohol consumption among veterans has been shown to be higher than that among the general population. Many veterans experience difficulties during the transition to post‐service life, and alcohol is used as a coping mechanism. Excessive alcohol use leads to a significant decrease in mental health, quality of life and social functioning, further exacerbating veteran's readjustment to civilian life after service.

**Objective:**

This study aimed to co‐design a transition programme to reduce problematic alcohol use. The objectives were to (1) understand which life domains need to be considered within programmes to support successful transition without harmful alcohol consumption and (2) co‐design a transition programme with New Zealand veterans (and service providers) to avoid harmful alcohol consumption.

**Design:**

This study offers a novel approach to the development of programmes to reduce excessive alcohol consumption by veterans through the use of a participatory design method. The study involved four co‐design workshops with veterans and Defence health professionals, in Aotearoa‐New Zealand, and abductive analysis of qualitative data. The analysis compared perspectives obtained from veterans and health professionals with existing well‐being and transition frameworks.

**Results:**

Findings supported recommendations within those frameworks for strategies to support mental, physical, social/family and spiritual well‐being, as well as finding meaningful work or employment. Themes emerged beyond those frameworks, including a need for programmes to manage loss of identity; lack of trust, scepticism and stigma; and a desire for connected records and networked services.

**Conclusions:**

The research offers practical recommendations for a co‐designed veteran well‐being ecosystem. This involved early prevention, in‐service elements and ongoing support through transition and in post‐service life. This was supported with the suggestion for a network of services that is promoted well and makes it easy for veterans to identify services that can increase their feeling of competence as they navigate transition.

**Patient or Public Contribution:**

This study used a co‐design process that engaged veterans and Defence health professionals in the design of a programme and programme elements that they would like to see for veterans like themselves or veterans they have encountered in practice.

## 1 Introduction

The military‐to‐civilian transition includes departure from the military, reorientation and adaptation to civilian life, and ideally, thriving as a civilian [1]. Transition can involve significant readjustment due to the difference between who someone knew themselves to be as a military person and who they are in civilian society [2]. Differences arise due to incongruence between individualistic societal values and collectivist military values [3, 4] and removal from a highly structured environment, containing routine, comradeship, loyalty, mutually dependent task performance and common purpose [5, 6]. Many veterans experience transition difficulties [3, 7, 8].

Alcohol consumption among veterans has been observed to be higher than that among the general population [9, 10]. In New Zealand, veterans were more likely to have ongoing drug and alcohol problems than the general population [11]. Similarly, in Australia, 40% of men who have served in the Defence Force reported exceeding the Australian Adult Alcohol Guideline [12] and 13% of members who have left full‐time regular service (both genders) met the criteria for an alcohol disorder in the last 12 months [13]. In the United States, a 10‐year, nationally representative longitudinal study of US veterans found more than 1 in 4 veterans consumed alcohol in at‐risk‐to‐excessive levels [9]. In the United Kingdom, veterans reported a significantly higher prevalence of alcohol misuse (11% vs. 6%) compared to non‐veterans [10]. These studies indicate the widespread prevalence of alcohol misuse within veteran populations across many nations. Alcohol consumption is common and accepted in the military environment and is used as a coping mechanism to regulate emotions and reduce anxiety [14, 15]. This socialisation or normalisation supports the use of alcohol as a coping mechanism [16] and deters recognition of alcohol problems and help‐seeking in veteran life [17]. Excessive alcohol use leads to a significant decrease in mental health, quality of life and social functioning [18], further exacerbating veteran's readjustment to civilian life after service [19].

Evidence suggests civilian alcohol reduction programmes are less suited to veterans, given their characteristics, transition experiences and factors that stimulate their alcohol use. Brief Alcohol Interventions, recommended by WHO for the general population, have a limited effect on veteran populations [20]. Researchers recommend tailored interventions for veterans to accommodate specific needs such as connection, peer support and identity loss [21, 22], holistic approaches to support veteran well‐being across multiple life domains [1] and cultural approaches to support Indigenous veterans [23]. Internationally, a range of services are commonly available to support veterans, including counselling, educational and employment assistance, disability compensation, physical and mental health services and substance abuse programmes [24]. In some countries, these services extend to legal and financial services, housing, home care and caregiver services [24]. Interventions are required to address the specific needs of veterans and their families, given the nuances of their experiences in the military and during the transition to civilian life.

This study offers a novel perspective on reducing veterans' alcohol use by co‐designing approaches together *with* them, rather than *for* them. Co‐design encourages participants to contribute their knowledge and skills as experts on their unique experiences [25]. An abductive analytical approach was taken to accommodate guiding frameworks for veteran and Indigenous health whilst investigating veterans' ideas for novel approaches to reduce alcohol consumption [26]. This manuscript begins by reviewing existing well‐being frameworks, then outlines the co‐design methodology employed and presents findings from the co‐design workshops. We conclude with contributions for veteran programme development from a co‐design lens and recommendations for further research to establish the acceptability and impact of the co‐designed strategies.

### 1.1 Well‐Being Frameworks for Veterans in Aotearoa—New Zealand

Frameworks have been conceptualised to guide the development of veteran transition programmes, covering multiple life domains considered necessary for successful transition. Karre et al. [1] synthesised multiple frameworks and subsequently compared the synthesised framework with data from transitioning veterans. This framework includes five domains: physical health, mental health, social, employment and financial. Physical health encompasses health‐supporting behaviours (such as eating healthily), avoidance of risky health behaviours (like substance abuse) and individual satisfaction with one's physical health. Mental health involves the experience of symptoms (anxiety and depression) and individual satisfaction with mental and emotional health. Social is characterised by social support and connection, as well as individual satisfaction with social networks and communities. Employment relates to the status achieved through work, including satisfaction with the type of work performed and the benefits received. Financial involves the ability to meet immediate and future financial needs. The Karre et al. [1] framework represents a holistic approach to veteran transition; however, being developed in the United States may not meet the needs of Aotearoa—New Zealand veterans.

Te Whare Tapa Whā (House of Four Walls) draws on indigenous New Zealand knowledge (kaupapa Māori) to create a health framework that advocates for a holistic approach to health and well‐being [27]. Although drawing on indigenous Māori knowledge, it has been employed for the benefit of Māori and non‐Māori alike, with the understanding that what is beneficial for Māori is beneficial for all. The four walls represent domains of health and well‐being, and all four need to be strong for sustained health and well‐being [27]. The four walls are taha hinengaro (mental well‐being), taha wairua (spiritual well‐being), taha tinana (physical well‐being) and taha whānau (social or family well‐being). A fifth domain is sometimes represented, being the connection to whenua (land/roots)—recognising the connection to one's origins [28]. Te whare tapa whā has been employed as a framework to help understand issues of prisoner well‐being [29], tobacco use [30], addiction [31], cancer patient recovery [32] and educational success [33]. It is an attempt to move beyond a western medical model of care, where much of the power sits with the health practitioner [34]. Instead, it more accurately reflects a Māori perspective that contextualises people in time and place and as part of a social system, whilst recognising and incorporating things that cannot be measured using a medical model to create a holistic view of health [35]. Purdy [34] provides a further explication of a contemporary application of te whare tapa whā to demonstrate its value in a health context from a Māori perspective.

### 1.2 Co‐Design

Behaviour change disciplines are increasingly underscoring the importance of a deep understanding of the people who are the focus of a programme. This involves the use of human‐centred or participatory design approaches, where solutions are created with people rather than for them to co‐create value [36, 37]. These approaches encourage individuals most closely involved with the issue to contribute to programme development as ‘experts of their own experience’ [25]. Processes can extend to other stakeholders, such as service providers and those with technical expertise. Participatory design methods have the potential to enhance outcomes, including adoption, satisfaction, retention and effectiveness [38]. Achieving optimal outcomes for veterans requires integrating the perspectives of veterans and their communities into the design of programmes that serve them [39].

This study aimed to co‐design a transition programme to reduce problematic alcohol use. The objectives were to (1) understand which life domains need to be considered within programmes to support successful transition without harmful alcohol consumption and (2) co‐design a transition programme with New Zealand veterans (and service providers) to avoid harmful alcohol consumption. The main contributions of this paper are veterans' perspectives on programme features they consider beneficial (or not), the identification of preferred and new programme features within life domains recommended by experts for successful transitions, and the conceptualisation of an ecosystem of programme/services to support veterans during transition to avoid problematic alcohol use.

## 2 Materials and Methods

This study involved veterans and Defence health professionals in New Zealand. The purpose was to co‐design programmes (or programme elements) for veterans to reduce the harmful effects of alcohol when individuals return to civilian life following service. This study was preceded by a systematic literature review, interviews with veterans and their whānau, and surveys to identify characteristics of NZ veterans vulnerable to alcohol misuse. These prior research activities informed this co‐design research, which sought to identify intervention points inside and outside of service to reduce the potential for harmful alcohol use. The research was approved by the University of Canterbury Ethics Committee [Ref: HREC 2023/89], Griffith University Ethics Committee (GU Ref No: 2023/873) and the NZ Defence Ethics Committee [Ref: Org Research 2023/22].

### 2.1 Participants

Participants were invited to attend co‐design workshops through social media, veterans' networks and researcher networks. Four workshops, each approximately 4 h long, were held from November 2023 to February 2024. Three workshops involved veterans, and one workshop involved Defence health professionals. Veteran participants were reimbursed for their time with $50 grocery or fuel vouchers, and Health professionals attended in paid work hours (no additional incentive was offered). The participant sample included 12 veterans and 7 Defence health professionals. The veterans were predominately male (83%), aged 28–73 years. The health professional group was predominantly male (57%) and comprised individuals aged 32–56 years. This study employed a convenience sampling method and did not aim to obtain a representative sample of veterans or health professionals. Instead, it sought to bring together information‐rich cases to provide rich and thick descriptions of veterans' experiences and preferences for programmes to support a transition to civilian life that does not involve alcohol misuse.

### 2.2 Procedure

**Figure 1:**
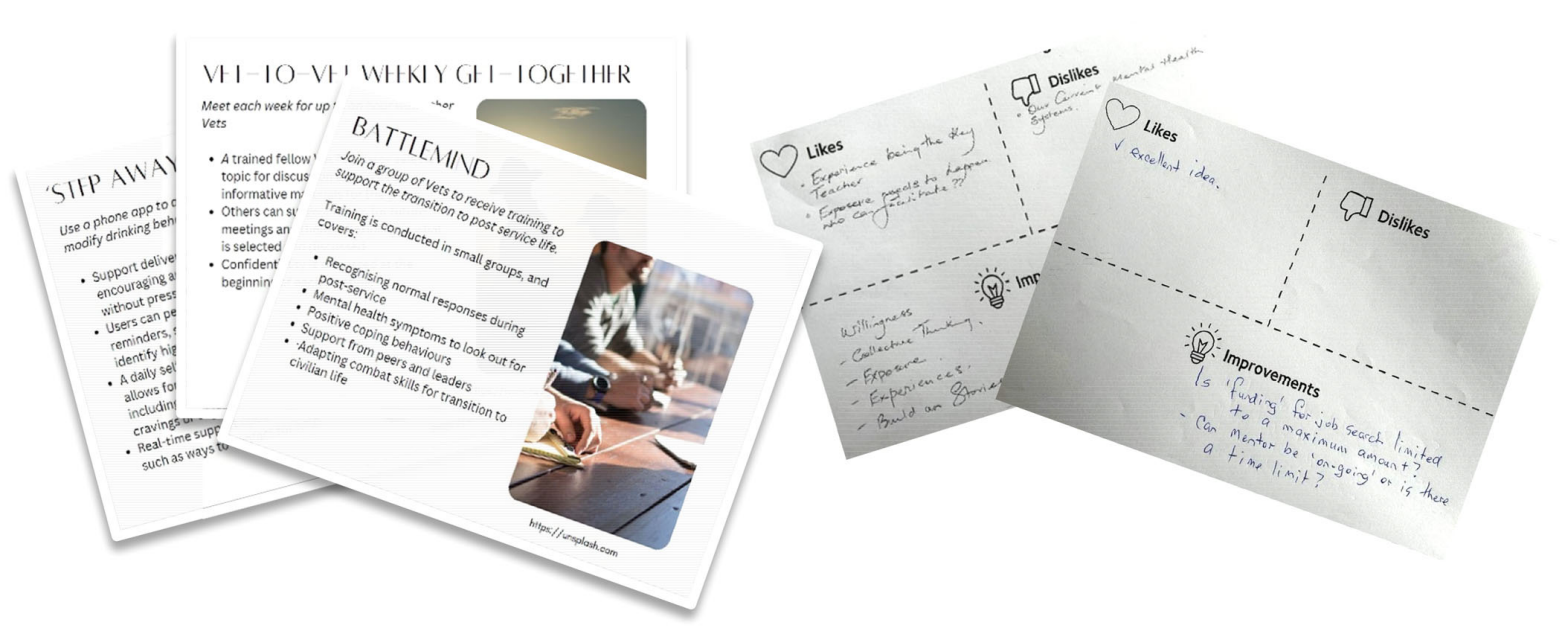
Stimulus card examples and feedback grids used in the first co‐design activity.

The workshops followed a sequential co‐design process [40] and involved activities that have been used in other co‐design workshops [41, 42]. Following a brief warm‐up activity, participants were given a series of handouts (stimuli) that described veteran alcohol harm reduction programmes. Each handout described a programme identified during the literature review in brief sentences, accompanied by an illustration (see Figure 1). Twelve programmes were described, which varied across dimensions of duration (brief/long), commitment (regular/occasional), facilitation (self‐paced/guided), interaction (online/in‐person) and content. Participants reviewed each programme individually, recording their responses on feedback grids that contained three feedback categories: like, dislike and improvement (see Figure 1). Participants then shared their feedback with the group, which encouraged them to think critically about programme strategies and prepared them to design new programme ideas.

**Figure 2:**
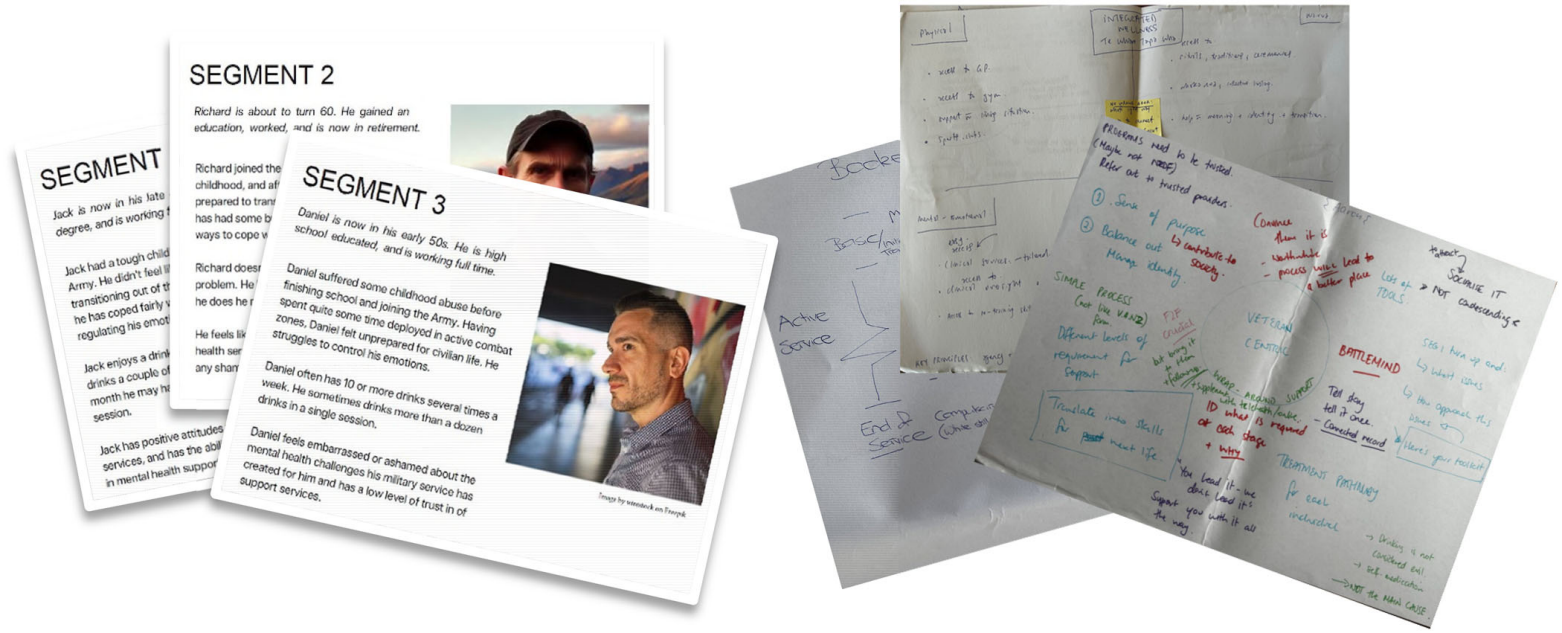
Segment personas examples and co‐design sheets from the final activity.

The final workshop task required participants, both veterans and health professionals, to generate ideas for a new programme they would like to see for veterans—veterans like themselves or those they have encountered in practice. They were also offered descriptions of three veteran personas, developed from the previous survey research, should they wish to design for one of those personas (see Figure 2). The programme design activities were conducted collaboratively in pairs or individually (if participants chose to) and were captured on large sheets of paper as words, flow diagrams, pictures or a combination of these (see Figure 2). Once new programme ideas had been captured, participants presented their ideas to the entire group and others in the group were encouraged to provide feedback or ask questions. The co‐design process was the same for veterans and Defence health professionals; however, a final step was added with health professionals, where they were asked to consider the feasibility and attractiveness of programme ideas from the veteran co‐design workshops.

Participants willingly engaged in each task, producing 216 feedback grids and 10 co‐designed solutions presented on large sheets representing ideas for new programmes. Discussions within the workshop about the feedback grids and co‐designed solutions were audio recorded and transcribed verbatim, producing 8 h and 39 min of audio data. Researchers also took field notes. These feedback grids, audio recordings, field notes and photographs of the co‐designed solution sheets represented the data for analysis.

### 2.3 Analysis

Content analysis was used to synthesise veterans' and health professionals' ideas for new programmes. An abductive approach was employed, which seeks insights based on existing knowledge whilst also reflecting on new empirical findings to modify and extend existing theories or frameworks in novel ways [43]. This involved exploring the data within existing well‐being frameworks [1, 27] by comparing and contrasting collected information with the framework to determine coherence or divergence. The analysis involved (1) data familiarisation (reviewing all textual data, photographs and audio recordings), (2) developing a coding frame based on the domains of the well‐being framework and (3) coding according to the coding frame whilst simultaneously remaining open to the identification of additional codes within the data. Investigators agreed on the initial coding framework, and then the first author performed the data coding. A second investigator reviewed the coding, and discussions occurred to refine and synthesise the themes, continuing until there was an agreement on how themes were situated within the well‐being frameworks and which elements represented new insights [44]. Differences in interpretation were mutually compatible rather than mutually exclusive [45]. This systematic analysis of the data against each theme and across the entire dataset generated a representation of the views of veterans and health professionals on how veterans' programmes could be constructed to contribute to well‐being and reduce alcohol consumption. The final step of the health professionals' workshop involved reviewing and considering the ideas from the veteran co‐design workshops. During this step, the health professionals indicated the ideas were acceptable and compatible with their professional knowledge of what may be helpful to transitioning veterans. During the analysis of data from all workshops, we found that the content of the discussions and ideas contributed by veterans and health professionals was broadly similar and compatible, rather than divergent. Therefore, themes were constructed from each of the four workshops jointly rather than separately.

## 3 Results

Throughout the co‐design sessions, participants acknowledged the difficulties many veterans have with alcohol consumption. They recognised the pervasiveness of drinking culture in the military (and afterwards) and the embedded nature of drinking within many ordinary activities.

Why do we drink alcohol? Because we ‘Wet the baby's head’… ‘Splice the mainbrace’… ‘I give you the king’ or even ‘Dutch courage’. Such expressions make alcohol a central acceptable part of Kiwi life.

Session #1 Co‐design solution #2

They emphasised that drinking in and of itself was not the problem; it was a form of ‘self‐medication’ and reflective of a struggle with other issues. They discussed the needs for veteran well‐being in a broad sense and co‐designed programmes that were holistic and stretched beyond the reduction of alcohol consumption in isolation. The well‐being domains of Durie [27] and Karre et al. [1] were evident, along with other themes. Existing and emergent themes were compared and contrasted, and then structured into three areas:

- Well‐being domains [27] and [1].
- Emergent themes:
  - Identity.
  - Trust, scepticism and stigma.
  - Connected records and networked services.
- A co‐designed veteran well‐being ecosystem.

**Figure 3:**
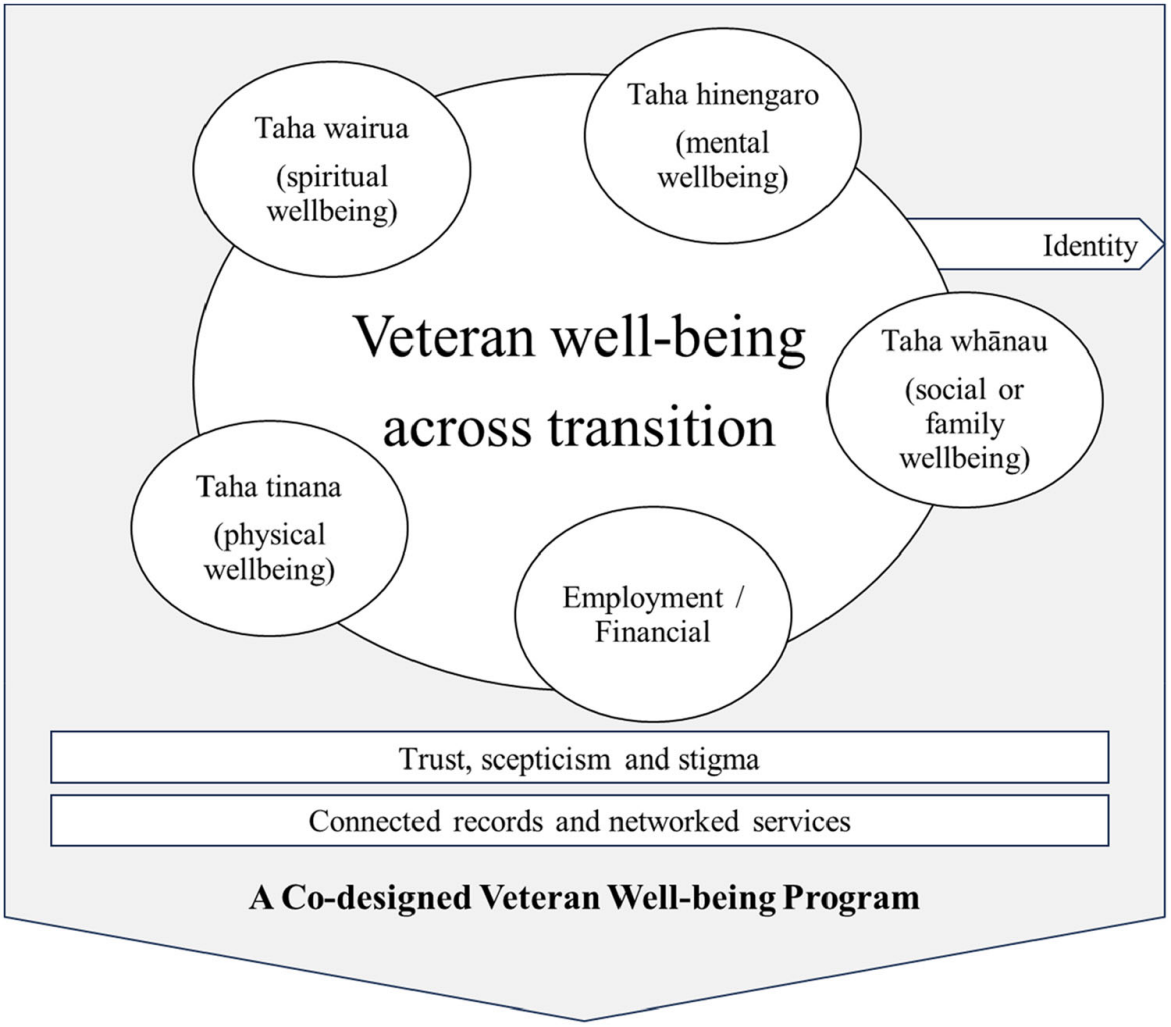
Synthesis of themes constructed during co‐design sessions.

Figure 3 visualises the synthesis of existing and emergent themes to illustrate how themes are connected. Next, each theme is discussed in detail, with examples from participants' contributions during the co‐design sessions.

### 3.1 Taha Hinengaro—Mental Well‐Being

Participants recognised the importance of mental well‐being for veterans, noting it can become destabilised during transition. They described the need for general mental health programmes, as well as programmes specifically related to veteran experiences, such as feelings of isolation, disengagement, being misunderstood and refusal to acknowledge excessive alcohol consumption or to seek help. They stated that programmes should help veterans establish agency within a supported environment and share their stories to build awareness of mental health challenges, provide acceptance and create cultural change. They spoke of the need to create opportunities for mental stimulation and development, as well as ‘challenge,’ to provide a meaningful and fulfilling post‐service life.

Service emphasis(es) non‐help‐seeking behaviour. [Use] media campaigns that are carefully constructed to not add more stigma…Respected gurus, maybe long form i.e., podcasts ‐ talking about mental health with a good host, anonymised media interview podcast with people from all domains of NZDF sharing stories as part of help‐seeking promotion. Language and tone must be very nuanced and not imply weakness or alienation.

Session #3 Co‐design solution #2

### 3.2 Taha Tinana—Physical Well‐Being

Physical well‐being was recognised as important to veterans post‐transition. Of interest, few participants mentioned physical harm caused by excessive alcohol consumption. However, they acknowledged that physical harms caused during service (physical injuries and/or lasting physical disability) contribute to the challenges of post‐service life. They suggested programmes should include treatment for physical health issues but frequently expressed a preference for programmes that included physical activities to support well‐being and as alternatives to activities that are more often associated with drinking. They referred to competitive or challenging activities that were familiar and aligned with their experiences during service, when building and maintaining physical fitness.

Outdoors adventures, hunting, fishing, diving, sailing as part of a recovery program…to maintain a level of self‐help, capability, comradeship, fear, and physical ability.

Session #3 Co‐design solution #1

### 3.3 Taha Whānau—Social or Family Well‐Being

Social connection—whether with whānau/family, friends, a prior service or veteran cohort, or the broader community—was described as a vital feature for programmes. Participants described the need for group activities and whānau/family services to support both the veteran and their whānau/family during transition, as well as to build connections that will support the veteran's post‐service life.

Must be able to incorporate spouse or whānau, not just the veteran.

Session #2; Feedback grid #3

Ability for families to meet each other and form social support community too. Support for them…more clinical support if required.

Session #4; Feedback grid #1

In addition, participants described a strong need for connection within the delivery of programmes, through face‐to‐face or personal support. Specifically, they preferred programmes delivered by people rather than online. When online options were discussed or suggested, they were supplementary to other programme elements or needed ‘*follow‐up or check‐in with a real person*’. Expert involvement (clinical help) or peer support was often mentioned, as was the need for tailoring to a veteran audience or a strong understanding of military life.

Needs a well‐trained professional with significant sociocultural familiarity. Not some wishy‐washy life coach who hasn't served talking about combat skills transitions etc…

Session #3; Feedback grid #4

### 3.4 Taha Wairua—Spiritual Well‐Being

Participants recognised the importance of cultural or spiritual elements for veteran well‐being. Co‐designed solutions included church or faith‐based involvement, or other cultural or spiritual elements that were not religion‐based. These included pastoral care, acknowledgement of care for the soul and performance of ceremonies.

Culture initiatives, not exclusively about alcohol, but addressing the need for status, meaning, involvement…church/faith community or Marae involvement/cultural involvement.

Session #4 Co‐design solution #2

Access to rituals, traditions, ceremonies.

Session #4; Feedback grid #3

Cultural, spiritual, church groups, music.

Session #2; Co‐design solution #3

### 3.5 Employment or Financial Well‐Being

Military transition involves separation from an organisation and the conclusion of a military career. Participants recognised the value of initiatives that assist veterans in establishing new careers or utilising their skills to contribute actively to society. They recognised that the early part of the transition might involve financial strain and funding for training activities may be required. They suggested a myriad of options to support re‐skilling, or entering a new career, from coaching on resume writing, career planning and identifying transferable military skills; study (both university, vocational, self‐study and within business education); short‐term job placement or voluntary roles; and networking, apprenticeships and work placements.

Start with 1:1 conversation with a transition coach in the transition period. Cover career planning, CV, skills, job hunting. Financial(ly) funded opportunities.

Session #4; Co‐design solution #1

Excellent! [referring to career assistance] It assists the Vet transition into Civvy life and arms them with the necessary skills and knowledge to make that happen.

Session #3; Feedback grid #1

### 3.6 Emergent Theme: Military Identity

Beyond employment or post‐service volunteer work, participants discussed ways to support veterans in managing their identity during the transition. This included links to the familiar (memorabilia, familiar structures and social groupings) and a caution that programmes that are too focused on practical strategies like career development (alone) do not recognise any loss and grief involved with a loss of military identity. Relatedly, participants spoke of the need for veterans to find purpose in new activities, particularly those that allow them to give service.

Interacting with other veterans is the key component that I like about this program. This would provide me with sociocultural familiarity that would help maintain a sense of self and shared identity…it needs to come at a time when the sense of self and connectedness begins to wane and reality sinks in and you realise you are just number and the military matches on without you.

Session #3; Feedback grid #4

### 3.7 Emergent Theme: Trust, Scepticism and Stigma

Participants emphasised the importance of trust within veteran programmes, given veterans can be vulnerable during the transition. Those delivering programmes should be socio‐culturally aware—familiar with military language, values and experiences. They spoke of distrust of doctors, perceptions that doctors do not consider holistic approaches and that resource constraints cause doctors to be dismissive. They disliked the word ‘resilience’ (and therefore resilience training) for service members, which carries through to veterans. Confidentiality was considered imperative, and participants expressed concerns that fear of lack of confidentiality would cause veterans to avoid engaging with programmes. The current definition of a veteran in New Zealand^1^ was considered to introduce inequities and add to concern about who has access to what levels of support. Together, these factors contributed to stigma or shame, as well as to avoidance of help‐seeking among veterans.

Clinical psychologist should have good sociocultural familiarity and ability to hold a space. Veterans need a space where they are all equal but accountable to a structure and well‐facilitated group support environment. Low stigma environment.

Session #3; Co‐design solution #2

Sadly the medical system lets people down in the sense of time allocated, information overload, [need to] extend the time, have purpose‐built programs of clinical resource that doesn't limit or cause a name and shame mentality.

Session #4; Feedback Grid #4

### 3.8 Emergent Theme: Connected Records and Networked Services

Despite concerns about confidentiality, participants expressed a desire for a system that would make veteran records available to programme providers. This would avoid the need for veterans to repeatedly validate their service records and reiterate their support needs, which compounds feelings of shame when asking for help. Furthermore, they felt veterans' prior service to their country should mean they are cared for post‐service—regardless of whether they met the current definition of a veteran. Having records more readily available would provide easier access to services. Coupled with this, they recognised that many helpful services are available, and opportunities exist to support veterans in finding and navigating this network of services.

Tell [your] story…tell it once…[a] connected record [and] treatment pathway for each individual…lots of tools

Session #1; Co‐design Solution #1

Promotion of programs available…promotion of services (RSA, Vet to Vet)… highlight groups and community organisations in departing members community/geographic region

Session #2; Co‐design Solution #2

### 3.9 A Co‐Designed Veteran Well‐Being Ecosystem

The themes described above, together with additional suggestions from the sessions, were synthesised to outline a programme or ecosystem of support services and programmes to support veterans' well‐being during the transition and reduce excessive alcohol consumption. The ecosystem was characterised by a through‐career (and beyond career) approach, the inclusion of tailored content appropriate for a veteran audience and a variety of activities to provide options—given that veterans may be at different stages of need or readiness during the transition. The characteristics of the proposed veteran well‐being ecosystem are now discussed.

Participants recognised that the moment of departure from the military might be too late to arm personnel with skills to enable them to adapt and resist turning to alcohol to deal with transition challenges. They suggested that activities should be introduced as early as basic military training, combined with regular check‐ins throughout the military career, before allocation to a ‘transition unit’ where they could access training and activities to prepare for civilian life. Participants suggested a range of services tailored to various veteran situations and preferences. They recognised some may already be offered, but stated they found them hard to find or were unaware of them. They suggested regular contact within the first 12–18 months, with options to remain connected to services beyond that period depending on the needs of the veteran.

Participants suggested several activities spanning the military career and post‐career (veteran) timeframe to support veteran transition and reduce alcohol consumption. Activities included mental and emotional resilience training (although preferably without using the term ‘resilience’), ideally delivered early in a military career. This, together with training and awareness about alcohol misuse delivered periodically throughout the military career, was proposed to change the drinking culture and support personnel to look out for themselves and others to prevent issues from developing. Participants suggested compulsory transition training or having a transition or career coach for each person to prepare them for civilian life. This coaching involved preparing for a new career, including CV preparation, identifying skills, assistance with job hunting and even financial support to facilitate these processes. While these services may have been available, most participants were unaware of them or did not feel they could utilise them.

For programme elements post‐service, participants suggested a suite of options for veterans to engage in. Almost all personnel suggested a mix of outdoor or sports activities, cultural activities, mentally stimulating activities (such as education or book clubs) and access to health or clinical services. Suggestions were also made for identity training—assisting personnel in finding meaning and purpose in post‐service life—and options that involve ‘giving back’ were suggested. The option of online services was suggested to retain an element of flexibility; however, the majority suggested programme elements that incorporate in‐person components, such as one‐to‐one sessions and small or large groups, with mentors, peers and professionals. The option for inclusion of family/whānau in activities was also considered important. It was recognised that some of these activities are currently available to veterans, but participants saw the need for a way to inform veterans of these services and connect veterans to them.

Across these activities, participants emphasised the need for tailored content and approaches. They felt that a ‘cookie‐cutter’ approach would not be suited to their needs. Familiarity with military customs, language and experiences was considered important to ensure engagement and to give confidence that the programmes would help veterans progress through the transition. Being able to ‘speak freely’ in ‘military lingo’ was necessary to ensure acceptance within group settings and prevent alienation. When experts or professionals were proposed as leaders of activities or within clinical settings, participants stated those experts need to be well‐trained to understand veterans' situations, experiences and ways of expressing themselves. Finally, many proposed activities were social in nature and aimed to replicate the team environment that exists in the military. Participants acknowledged that the team environment is lacking post‐service, which is something familiar to veterans and could help them feel more comfortable in civilian life.

## 4 Discussion

This study adopted a participatory design method to understand veterans' preferences for programmes to support their well‐being during transition and, in turn, reduce excessive alcohol consumption. A co‐design method was employed to explore and develop new ideas for programmes *with* veterans and health professionals rather than developing *for* them. Assisting veterans before, during and after transition requires ‘meeting them where they are’ and offering tailored strategies that respect the differences between veterans [47]. Indeed, participants heavily criticised programmes and experiences that did not cater for individual circumstances or show a socio‐cultural understanding of the veteran space.

This study uncovered views on well‐being during and beyond transition, as well as perspectives on programmes previously implemented for veterans, and developed new programme ideas through three co‐design sessions with veterans and one with Defence health professionals. The following significant insights were revealed:

1. Confirmation and new empirical evidence aligned with previous conceptualisations of veteran well‐being during transition [1] and health and well‐being in Aotearoa—New Zealand [27].
2. Identification of important themes for veteran programmes, including loss of identity, trust, scepticism and stigma, and the need for connected and networked services.
3. A co‐designed ecosystem of services for veterans to support well‐being during the transition to reduce excessive alcohol consumption.

### 4.1 Contribution to Veteran Transition Literature

This study provides empirical evidence supporting the military‐to‐civilian veteran well‐being framework [1] and prior research that takes a holistic view of veteran transition [48]. It also supports the utility of integrating Māori knowledge of health and well‐being [27] for veterans in Aotearoa—New Zealand. The research found support for the importance of physical well‐being (desire for physical activities and challenges), mental well‐being (clinical services), social and family well‐being (preference for peer and social connection and family involvement), spiritual well‐being (desire for cultural activities), and employment and financial stability (career coaching). These findings align with the international literature, which indicates that veterans face challenges across a range of life domains. These include employment, education and financial challenges, particularly early in post‐service life, whilst mental health, physical health and social challenges can persist over the long term [49]. The literature indicates that many nations offer education and employment assistance, counselling, physical and mental health services and substance abuse programmes [24]. Furthermore, common components analysis has been used to synthesise programme elements that support successful military‐to‐civilian transitions [50] into life domains, such as those proposed by Karre et al. [1], which bear similarity to the health and well‐being domains of Durie [27].

In addition to the constructs within these frameworks, this study identified emergent themes of identity loss occurring after separation from the military [51] and an expressed need for assistance in managing identity transformation [52]. Furthermore, the study echoed other research showing veterans experience stigma or shame associated with seeking help [53] and have a low level of trust in medical and several other support providers [54]. Interestingly, we observed a strong desire for networked services that rely on a high level of trust when records are readily available to multiple providers.

### 4.2 An Ecosystem of Services for Transitioning Veterans

**Figure 4:**
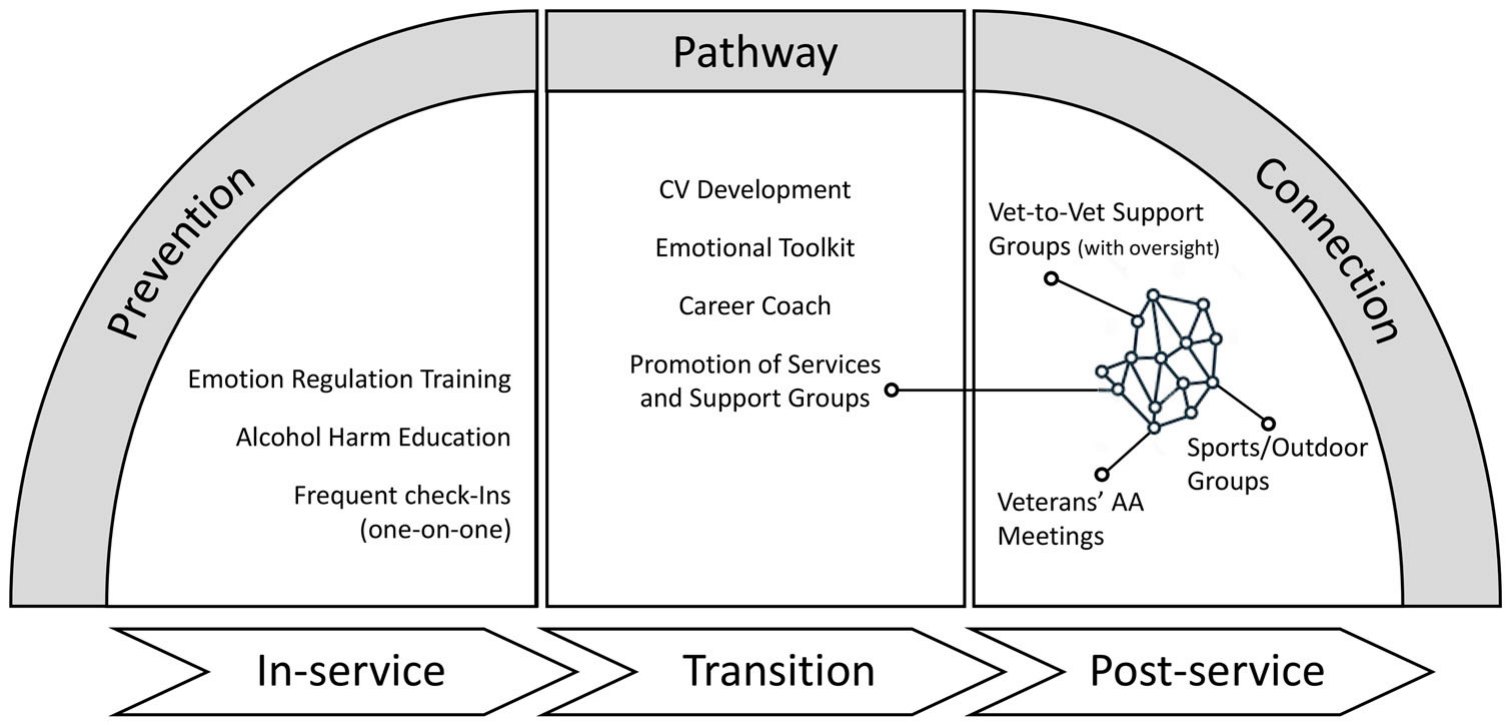
Consolidation of co‐design ideas into an ecosystem of services for veteran well‐being.

This study identified the potential for an ecosystem of services to support veterans during transition. This involved early prevention, in‐service training elements, and ongoing support throughout transition and post‐service life. These are summarised in Figure 4, which shows the consolidated set of co‐designed programme ideas for veteran well‐being aimed at reducing excessive alcohol use.

Suggestions for strategies include early prevention, recognising the importance of emotion regulation training and alcohol harm education in early career, and maintaining this knowledge and awareness through regular touchpoints. Additional suggestions covered the *transition window* (the period approaching separation and immediately afterwards), which focuses on recognising transferable skills and developing career or occupational options (including volunteer or purposeful non‐paid activities for those not seeking employment). Finally, the last set of suggestions involved establishing and maintaining *connections in veteran life through various activities, involving a wide range of connections* with peers, family and new social groups. These suggestions align with a holistic concept of veteran well‐being and could underpin the development of a veteran ecosystem to support veterans to function and thrive as part of civilian society, beyond solely alleviating the health or societal burdens of excessive alcohol consumption. Additionally, a positive approach to well‐being does not stigmatise, which contributes to low help‐seeking by veterans. An ecosystem approach prepares veterans for transition and enables veterans to dip in and out of the network in post‐service life, allowing them to find parts of the network that support their needs at particular times and stages of transition.

### 4.3 Theoretical Insights

A holistic approach to veteran well‐being during transition shifts the focus from problematising excessive alcohol consumption to striving for well‐being across multiple domains to reduce reliance on alcohol as a regulator of emotions [14] or as self‐medication [55]. This study extends that thinking, finding trust, scepticism and stigma to be associated with some veteran services and providers of alcohol treatments, and through a preference for programme elements and networked approaches to provide a more positive view to support veterans with alcohol misuse—through striving for holistic well‐being.

### 4.4 Methodological Insights

This study employed a participatory design method known as co‐design [40]. Co‐design (and other participatory design methods) are frequently employed by researchers because they provide a means to increase engagement with end‐users and enhance the acceptability and effectiveness of solutions [56, 57]. This paper makes a contribution by demonstrating the use of co‐design to develop an approach to veteran well‐being during the transition, with programme features and system characteristics, which participants have suggested would reduce excessive alcohol consumption. In the veteran domain, participatory research approaches are gaining traction [39]; however, co‐design has been more commonly used to develop service screening methods [58] or information technology solutions [59, 60], than programmes or service ecosystems. Furthermore, some co‐design attempts have involved a majority of experts and only some veteran input [61] rather than adopting a deeper participatory approach that places the end‐user in the driver's seat during the design of programmes intended for them. Research has contributed to the understanding of excessive alcohol use by veterans [14] and veterans' experiences during the transition [62], which is then used to underpin the design of services or policies to support veterans. However, even though these efforts are guided by insights into veterans' experiences, experts often act as solution creators [63]. This study demonstrates how veterans can be supported and empowered to create innovative solutions through co‐design.

### 4.5 Practical Insights

This study highlights the impact of transition from military service on post‐service well‐being for a veteran and their family/whānau. Taking a career‐spanning view of transition and a holistic approach to services was suggested to prevent issues arising with alcohol use before separation and ensure ongoing support as a veteran. This involves instilling positive behaviours early in a military career through alcohol awareness and emotional regulation training and ensuring regular touchpoints to maintain awareness and ongoing benefits from training. A holistic approach encourages the involvement of others, such as family and peers, in elements of transition, providing social support that veterans are accustomed to from team‐based military structures. This approach also fosters the establishment of new, purposeful connections within civilian society. For example, volunteering within sports clubs continues the association with a physically active lifestyle, while also building on the nature of service life by providing service to the sporting club.

Further practical implications relate to the creation of a veteran network to support transition. The focus should be on developing and connecting veterans to a network of new and existing services, making it easier for them to obtain support. Preferred strategies identified in this study include online options for flexibility and younger veterans, promoting support groups, referrals to clinical services and connections with employers or volunteer groups. While in service, personnel have access to the military health system, which provides care and referrals through the system [64]. Upon separation, personnel are required to relearn and utilise the civilian healthcare system and are often reluctant to seek help [53]. A network of services that is promoted well and makes it easy for veterans to identify services can increase feelings of competence as veterans navigate the transition.

### 4.6 Limitations and Future Research Directions

The current study was conducted in one country (Aotearoa—New Zealand). Future research may explore whether veterans in other nations, with different military customs, organisational policies and procedures and veteran transition services, have similar or divergent views on how to support veterans during the transition. In New Zealand, veterans are only classified as such if they have undertaken qualifying operational service. Therefore, many ex‐service persons are not classified as veterans. Suggestions for veteran programmes may differ in countries that have other definitions of veterans.

This study generated valuable insights using a single research method, yielding rich insights and innovative programme suggestions; however, the co‐design process has limitations that need to be acknowledged. The stimuli presented to participants represented programmes that had been previously implemented but could not encompass all possible programmes. Therefore, this study may have benefited from the inclusion of an even broader range of programmes for critique and inspiration. This study adopts a qualitative approach, focusing on generating an in‐depth understanding of participants' lived experiences [65] and elucidating their contributions to the design of strategies for veteran well‐being. Akin to other qualitative research, it involved a sample size large enough to generate ‘new and richly textured understanding’ but small enough to enable ‘deep, case‐oriented analysis’ [66] (p. 183). Further research is required to determine the transferability or comparability of these findings to other settings, populations and locations. A strength of this study was the inclusion of veterans and Defence health professionals. However, future research could extend this to in‐service members approaching transition, as well as to family/whānau, to generate further ideas for programmes. Further co‐design sessions or other design methods could be used to add greater details to programme ideas.

This study did not attempt to evaluate the acceptability or effectiveness of the suggested programme ideas. Quantitative methods such as surveys with a broader sample of veterans could confirm the acceptability of programme ideas generated by this study. Future research is needed to empirically assess the impact of the veteran programmes designed in this study. Given that there were several programme ideas nested across time, this would require a systematic evaluation of these ideas, potentially following personnel through transition, to understand whether a greater benefit is achieved when veterans have access to multiple services across a broad transition timeframe.

## 5 Conclusion

Supporting veterans to reduce excessive alcohol consumption requires taking a holistic view of veteran well‐being. This may include programme elements focusing on alcohol; however, in this study, veterans envisioned a broader set of programme elements to contribute to physical, mental, spiritual and social well‐being, as well as employment and financial stability. They suggested early prevention whilst in‐service, services during transition and throughout post‐service life. Furthermore, activities are needed to support identity transition, avoid stigmatising individuals and make identifying and accessing services easy. Veterans expressed a desire for an ecosystem that provides a variety of services to support their well‐being as they transition into and navigate post‐service life, enabling them to adapt and thrive as veterans.

## Author Contributions

**Julia Carins:** conceptualisation, funding acquisition, methodology, formal analysis, writing – original draft, investigation. **Ann‐Marie Kennedy:** conceptualisation, funding acquisition, methodology, formal analysis, writing – original draft, project administration, investigation. **Ekant Veer:** conceptualisation, funding acquisition, methodology, writing – review and editing.

## Conflicts of Interest

The authors declare no conflicts of interest.

## Data Availability

The anonymised data that support the findings of this study are available on reasonable request from the corresponding author. The data are not publicly available due to privacy and ethical restrictions.

## Endnotes

1 Those who have had any service in the New Zealand armed forces before 1 April 1974, and those with ‘qualifying operational service’ after that date. Qualifying operational service covers service at a time of war or in deployments overseas, where a ministerial declaration has confirmed significant risk of harm—New Zealand Defence Force [46].

## References

[1] J. K. Karre , D. F. Perkins , N. R. Morgan , et al., “What Do Successful Military‐to‐Civilian Transitions Look Like? A Revised Framework and a New Conceptual Model for Assessing Veteran Well‐Being,” Armed Forces & Society, ahead of print, January 9, 2024, 10.1177/0095327X231216678.

[2] E. Binks and S. Cambridge , “The Transition Experiences of British Military Veterans,” Political Psychology 39, no. 1 (2018): 125–142.

[3] A. Barnett , M. Savic , D. Forbes , et al., “Transitioning to Civilian Life: The Importance of Social Group Engagement and Identity Among Australian Defence Force Veterans,” Australian and New Zealand Journal of Psychiatry 56, no. 8 (2022): 1025–1033. 34541871 10.1177/00048674211046894

[4] S. Shepherd , D. K. Sherman , A. MacLean , and A. C. Kay , “The Challenges of Military Veterans in Their Transition to the Workplace: A Call for Integrating Basic and Applied Psychological Science,” Perspectives on Psychological Science 16, no. 3 (2021): 590–613. 33316201 10.1177/1745691620953096

[5] L. Cooper , N. Caddick , L. Godier , A. Cooper , and M. Fossey , “Transition From the Military Into Civilian Life: An Exploration of Cultural Competence,” Armed Forces & Society 44, no. 1 (2018): 156–177.

[6] W. H. McCormick , J. M. Currier , S. L. Isaak , et al., “Military Culture and Post‐Military Transitioning Among Veterans: A Qualitative Analysis,” Journal of Veterans Studies 4, no. 2 (2019): 288.

[7] D. McBride , A. Samaranayaka , A. Richardson , et al., “Factors Associated With Self‐Reported Health Among New Zealand Military Veterans: A Cross‐Sectional Study,” BMJ open 12, no. 5 (2022): e056916. 10.1136/bmjopen-2021-056916 PMC9134175 35613796

[8] D. F. Perkins , K. R. Aronson , N. R. Morgan , et al., “Veterans' Use of Programs and Services as They Transition to Civilian Life: Baseline Assessment for the Veteran Metrics Initiative,” Journal of Social Service Research 46, no. 2 (2020): 241–255, 10.1080/01488376.2018.1546259.

[9] P. J. Na , J. Montalvo‐Ortiz , I. Petrakis , et al., “Trajectories of Alcohol Consumption in US Military Veterans: Results From a 10‐Year Population‐Based Longitudinal Study,” Drug and Alcohol Dependence 246 (2023): 109833. 36963160 10.1016/j.drugalcdep.2023.109833 PMC10811960

[10] R. Rhead , D. MacManus , M. Jones , N. Greenberg , N. T. Fear , and L. Goodwin , “Mental Health Disorders and Alcohol Misuse Among UK Military Veterans and the General Population: A Comparison Study,” Psychological Medicine 52, no. 2 (2022): 292–302. 32777197 10.1017/S0033291720001944

[11] B. Cox , D. McBride , J. Broughton , and D. Tong , “Health Conditions in a Cohort of New Zealand Vietnam Veterans: Hospital Admissions Between 1988 and 2009,” BMJ Open 5, no. 12 (2015): e008409. 10.1136/bmjopen-2015-008409 PMC4679881 26656012

[12] AIHW . 2023. Health of Veterans, https://www.aihw.gov.au/reports/veterans/health-of-veterans.

[13] M. Van Hooff , E. Lawrence‐Wood , S. Hodson , et al. 2018. Mental Health Prevalence, Mental Health and Wellbeing Transition Study.

[14] A. K. Osborne , G. Wilson‐Menzfeld , G. McGill , and M. D. Kiernan , “Military Service and Alcohol Use: A Systematic Narrative Review,” Occupational Medicine 72, no. 5 (2022): 313–323, 10.1093/occmed/kqac045. 35674143 PMC9272263

[15] C. M. Young , E. R. Pedersen , A. D. Pearson , and C. Neighbors , “Drinking to Cope Moderates the Efficacy of Changing Veteran Drinking Norms as a Strategy for Reducing Drinking and Alcohol‐Related Problems Among U.S. Veterans,” Psychology of Addictive Behaviors 32, no. 2 (2018): 213–223, 10.1037/adb0000347. 29369674 PMC6863167

[16] C. D. Mohr , C. T. McCabe , S. N. Haverly , L. B. Hammer , and K. F. Carlson , “Drinking Motives and Alcohol Use: The SERVe Study of US Current and Former Service Members,” Journal of Studies on Alcohol and Drugs 79, no. 1 (2018): 79–87. 29227235 10.15288/jsad.2018.79.79 PMC5894860

[17] A. Cardow , J.‐S. Imbeau , B. W. Apiata , and J. Martin , “Leave No One Behind? Transitioning From the Military to Civilian Life in New Zealand,” Journal of Management & Organization 30, no. 2 (2021): 368–385.

[18] J. Levola , M. Aalto , A. Holopainen , A. Cieza , and T. Pitkänen , “Health‐Related Quality of Life in Alcohol Dependence: A Systematic Literature Review With a Specific Focus on the Role of Depression and Other Psychopathology,” Nordic Journal of Psychiatry 68, no. 6 (2014): 369–384. 24228776 10.3109/08039488.2013.852242

[19] A. C. Carter , C. Capone , and E. Eaton Short , “Co‐Occurring Posttraumatic Stress Disorder and Alcohol Use Disorders in Veteran Populations,” Journal of Dual Diagnosis 7, no. 4 (2011): 285–299, 10.1080/15504263.2011.620453. 23087599 PMC3474251

[20] S. Wigham , A. Bauer , S. Robalino , J. Ferguson , A. Burke , and D. Newbury‐Birch , “A Systematic Review of the Effectiveness of Alcohol Brief Interventions for the UK Military Personnel Moving Back to Civilian Life,” BMJ Military Health 163 (2017): 242–250. 10.1136/jramc-2016-000712 28320916

[21] D. M. Blonigen , B. Harris‐Olenak , E. Kuhn , et al., “Using Peers to Increase Veterans' Engagement in a Smartphone Application for Unhealthy Alcohol Use: A Pilot Study of Acceptability and Utility,” Psychology of Addictive Behaviors 35, no. 7 (2021): 829–839. 32597665 10.1037/adb0000598 PMC7769861

[22] K. M. Verkamp , “From Warrior Ethos to Obscurity: Veteran Reintegration Literature Review,” Journal for Nurse Practitioners 17, no. 5 (2021): 564–569.

[23] D. Wilson , E. Moloney , J. M. Parr , C. Aspinall , and J. Slark , “Creating an Indigenous Māori‐Centred Model of Relational Health: A Literature Review of Māori Models of Health,” Journal of Clinical Nursing 30, no. 23–24 (2021): 3539–3555. 34046956 10.1111/jocn.15859 PMC8597078

[24] M. Fossey , R. Lazier , M. N. Lewis , N. Williamson , and N. Caddick , “Military‐to‐Civilian Transition Policies, Processes, and Program Efforts,” in *Military Veteran Reintegration* (Elsevier, 2019), 51–74.

[25] E. B.‐N. Sanders and P. J. Stappers , “Co‐Creation and the New Landscapes of Design,” CoDesign 4, no. 1 (2008): 5–18.

[26] E. Hurley , T. Dietrich , and S. Rundle‐Thiele , “Integrating Theory in Co‐Design: An Abductive Approach,” Australasian Marketing Journal 29, no. 1 (2021): 66–77.

[27] M. Durie , Whaiora: Māori Health Development (Oxford University Press, 1994).

[28] H. Moewaka Barnes and T. McCreanor , “Colonisation, Hauora and Whenua in Aotearoa,” Journal of the Royal Society of New Zealand 49 (2019): 19–33.

[29] R. Hazou , S. Woodland , and P. Ilgenfritz , “Performing Te Whare Tapa Whā: Building on Cultural Rights to Decolonise Prison Theatre Practice,” Research in Drama Education: The Journal of Applied Theatre and Performance 26, no. 3 (2021): 494–510.

[30] M. Glover , “Descriptions of Depression Among a Sample of Maori Smokers,” New Zealand Journal of Psychology 34, no. 1 (2005): 4–12.

[31] D. Sculley , “A Living Curriculum: Interweaving Te Whare Tapa Whā, Model of Māori Holistic Health and Wairua, Into Postgraduate Mental Health and Addictions Nursing,” Whitireia Journal of Nursing, Health and Social Services 30 (2023): 29–35.

[32] R. Peniamina , C. Davies , L. Moata'ane , et al. 2021. Food, Nutrition and Cancer: Perspectives and Experiences of New Zealand Cancer Survivors. 34788269

[33] J. Higgins and S. Goodall , “Transforming the Wellbeing Focus in Education: A Document Analysis of Policy in Aotearoa New Zealand,” International Journal of Qualitative Studies on Health and Well‐being 16, no. 1 (2021): 1879370. 33525996 10.1080/17482631.2021.1879370 PMC8725755

[34] S. C. Purdy , “Communication Research in the Context of Te Whare Tapa Whā Model of Health,” International Journal of Speech‐Language Pathology 22, no. 3 (2020): 281–289. 32686594 10.1080/17549507.2020.1768288

[35] T. Rochford , “Whare Tapa Wha: A Mäori Model of a Unified Theory of Health,” Journal of Primary Prevention 25, no. 1 (2004): 41–57.

[36] C. Domegan , K. Collins , M. Stead , P. McHugh , and T. Hughes , “Value Co‐Creation in Social Marketing: Functional or Fanciful?,” Journal of Social Marketing 3, no. 3 (2013): 239–256.

[37] S. Rundle‐Thiele , T. Dietrich , and J. Carins , “CBE: A Framework to Guide the Application of Marketing to Behavior Change,” Social Marketing Quarterly 27, no. 3 (2021): 175–194.

[38] T. J. Willmott , A. Mathew , E. Luck , et al., “Participatory Design Application in Obesity Prevention Targeting Young Adults and Adolescents: A Mixed‐Methods Systematic Scoping Review Protocol,” Systematic Reviews 11, no. 1 (2022): 51. 35317866 10.1186/s13643-022-01900-z PMC8939071

[39] Z. Franco , K. Hooyer , L. Ruffalo , R. A. F.‐H. Fung , M. Flower , and J. Whittle , “Foreword to the Second Volume of the Special Issue on Veteran Community Engagement,” Journal of Humanistic Psychology 63, no. 6 (2023): 735–743, 10.1177/00221678231204932.

[40] J. Trischler , T. Dietrich , and S. Rundle‐Thiele , “Co‐Design: From Expert‐ to User‐Driven Ideas in Public Service Design,” Public Management Review 21, no. 11 (2019): 1595–1619.

[41] J. Carins and S. Bogomolova , “Co‐Designing a Community‐Wide Approach to Encouraging Healthier Food Choices,” Appetite 162 (2021): 105167. 33596438 10.1016/j.appet.2021.105167

[42] S. Rundle‐Thiele , T. J. Willmott , N. McKillop , P. Saleme Ruiz , and A. Kitunen . 2023. Young Voices United: Co‐Designing a Place‐Based Youth‐Led Sexual and Violence Abuse Prevention Approach for One Australian Community. *Safer Communities*.

[43] S. Timmermans and I. Tavory , “Theory Construction in Qualitative Research: From Grounded Theory to Abductive Analysis,” Sociological Theory 30, no. 3 (2012): 167–186.

[44] M. Schreier , Qualitative Content Analysis in Practice, 1st ed. (SAGE Publications Inc, 2012), 10.4135/9781529682571.

[45] R. W. Belk , M. Wallendorf , and J. F. Sherry, Jr. , “The Sacred and the Profane in Consumer Behavior: Theodicy on the Odyssey,” Journal of Consumer Research 16, no. 1 (1989): 1–38.

[46] New Zealand Defence Force . 2018. *The Veteran Rehabilitation Strategy*.

[47] Z. Franco , K. Hooyer , L. Ruffalo , and R. A. Frey‐Ho Fung , “Veterans Health and Well‐Being—Collaborative Research Approaches: Toward Veteran Community Engagement,” Journal of Humanistic Psychology 61, no. 3 (2021): 287–312.

[48] D. Pedlar , J. M. Thompson , and C. A. Castro , Military‐to‐Civilian Transition Theories and Frameworks. In *Military Veteran Reintegration* (Elsevier, 2019), 21–50.

[49] Veteran Evaluation & Research Applications Network . 2025. *Successful Transition Domains Over Time*, veternetwork.pse.edu.

[50] D. F. Perkins , K. J. McCarthy , N. R. Morgan , et al., “Translation of a Longitudinal Survey of Veterans' Well‐Being Into Action by a Research‐Practice‐Policy Partnership,” Frontiers in Public Health 12 (2024): 1346057. 39776475 10.3389/fpubh.2024.1346057 PMC11703825

[51] M. Flack and L. Kite , “Transition From Military to Civilian: Identity, Social Connectedness, and Veteran Wellbeing,” PloS one 16, no. 12 (2021): e0261634. 34936679 10.1371/journal.pone.0261634 PMC8694481

[52] B. C. Semaan , L. M. Britton , and B. Dosono . 2016. Transition Resilience With ICTs: “Identity Awareness” in Veteran Re‐Integration. Proceedings of the 2016 CHI Conference on Human Factors in Computing Systems.

[53] M. D. Kiernan , A. Osbourne , G. McGill , P. Jane Greaves , G. Wilson , and M. Hill , “Are Veterans Different? Understanding Veterans' Help‐Seeking Behaviour for Alcohol Problems,” Health & Social Care in the Community 26, no. 5 (2018): 725–733. 10.1111/hsc.12585 29851155

[54] R. Randles and A. Finnegan , “Veteran Help‐Seeking Behaviour for Mental Health Issues: A Systematic Review,” BMJ Military Health 168, no. 1 (2022): 99–104. 34253643 10.1136/bmjmilitary-2021-001903

[55] A. S. London , J. M. Wilmoth , W. J. Oliver , and J. A. Hausauer , “The Influence of Military Service Experiences on Current and Daily Drinking,” Substance Use & Misuse 55, no. 8 (2020): 1288–1299. 32167849 10.1080/10826084.2020.1735438

[56] P. David , S. Rundle‐Thiele , B. Pang , K. Knox , J. Parkinson , and F. Hussenoeder , “Engaging the Dog Owner Community in the Design of an Effective Koala Aversion Program,” Social Marketing Quarterly 25, no. 1 (2019): 55–68.

[57] J. Talevski , S. T. Kulnik , R. L. Jessup , R. Falls , N. Cvetanovska , and A. Beauchamp , “Use of Co‐Design Methodology in the Development of Cardiovascular Disease Secondary Prevention Interventions: A Scoping Review,” Health Expectations 26, no. 1 (2023): 16–29. 36366855 10.1111/hex.13633 PMC9854329

[58] F. Goodyear‐Smith , M. Darragh , and J. Warren , “VeCHAT: A Proof‐of‐Concept Study on Screening and Managing Veterans' Mental Health and Wellbeing,” Journal of Primary Health Care 13, no. 1 (2021): 75–83. 33785114 10.1071/HC20070

[59] H. M. LaMonica , T. A. Davenport , J. Burns , et al., “Technology‐Enabled Mental Health Service Reform for Open Arms–Veterans and Families Counselling: Participatory Design Study,” JMIR Formative Research 3, no. 3 (2019): e13662. 31538937 10.2196/13662 PMC6754687

[60] J. H.‐M. Lu , C. Corrales , and B. Semaan . 2019. Designing for Separation: Participatory Design With Military Veterans. *iConference 2019 Proceedings*.

[61] A. K. Osborne , G. McGill , P. J. Greaves , and M. D. Kiernan , “Developing an Integrated Model of Care for Veterans With Alcohol Problems,” International Journal of Integrated Care 22, no. 1 (2022): 1–13, 10.5334/ijic.5500. PMC8855732 35282154

[62] S. Rose , E. VanDenKerkhof , and M. Schaub , “Determinants of Successful Transition Literature Review,” Journal of Military, Veteran and Family Health 4, no. 1 (2018): 90–99.

[63] A. Sarwar and P. T. Fraser , “Explanations in Design Thinking: New Directions for an Obfuscated Field,” She Ji 5, no. 4 (2019): 343–355.

[64] M. Bricknell and P. Cain , “Understanding the Whole of Military Health Systems: The Defence Healthcare Cycle,” RUSI Journal 165, no. 3 (2020): 40–49.

[65] S. A. Mthuli , F. Ruffin , and N. Singh , “‘Define, Explain, Justify, Apply’(DEJA): An Analytic Tool for Guiding Qualitative Research Sample Size,” International Journal of Social Research Methodology 25, no. 6 (2022): 809–821.

[66] M. Sandelowski , “Sample Size in Qualitative Research,” Research in Nursing & Health 18, no. 2 (1995): 179–183. 7899572 10.1002/nur.4770180211

